# Smoking among hospitalized patients: A multi-hospital cross-sectional study of a widely neglected problem

**DOI:** 10.18332/tid/92927

**Published:** 2018-07-30

**Authors:** Cristina Martínez, Marcela Fu, Yolanda Castellano1, Anna Riccobene, Paz Fernández, Sandra Cabrera, Eva Gavilan, Ariadna Feliu, Montse Puig-Llobet, Pilar Fuster, Jose María Martínez-Sánchez, Javier Montes, Joan Maria Estrada, Carmen Moreno, Anna Falcó-Pegueroles, Jordi Galimany, Cecilia Brando, Rosa Suñer-Soler, Anna Capsada, Esteve Fernández, y Grupo de Coordinadores de la Red Catalana de Hospitales sin Humo (XCHsF)

**Affiliations:** 1 Tobacco Control Unit, Cancer Control and Prevention Programme, Institut Català d’Oncologia-ICO-IDIBELL, Barcelona, Spain; 2 School of Nursing, Faculty of Medicine and Health Sciences, University of Barcelona, Barcelona, Spain; 3 School of Medicine, Department of Clinical Sciences, Universitat de Barcelona, Barcelona, Spain; 4 Nursing Research Unit, Institut Català d’Oncologia-ICO, Barcelona, Spain; 5 Medicine and Health Sciences School, Universitat Internacional de Catalunya, Barcelona, Spain; 6 Department of Nursing Science, Gimbernat School, Barcelona, Spain; 7 Department of Nursing Science, University of Girona, Girona, Spain; 8 Fundació Althaia, Barcelona, Spain

**Keywords:** smoking, tobacco, epidemiology, policy, health services

## Abstract

**INTRODUCTION:**

A comprehensive smoking ban was recently enacted for acute-care hospital campuses in Spain. The aim of this study was to assess the prevalence and patterns of smoking among inpatients before and during hospitalization.

**METHODS:**

Multi-center cross-sectional study was conducted in 13 hospitals in the province of Barcelona, Spain from May 2014 to May 2015. Participants were adults who provided informed consent. The sample size was calculated to be representative of each hospital (prevalence 29.4%, precision ± 5%, error 5%). We approached 1228 subjects, 888 accepted to participate and 170 were replaced (were not available or declined to participate). Final sample comprised 1047 subjects. We used a computer-assisted personal interview system to collect data, including sociodemographic variables and use of tobacco before and during hospitalization. Smoking status was validated with exhaled carbon monoxide. We calculated overall tobacco prevalence and investigated associations with participant and center characteristics. We performed multiple polytomous and multilevel logistic regression analyses to estimate odds ratios (ORs) and 95% confidence intervals (CIs), with adjustments for potential confounders.

**RESULTS:**

In all, 20.5% (95% CI: 18.1–23.0) of hospitalized patients were smokers. Smoking was most common among men (aOR=7.47; 95% CI: 4.88–11.43), young age groups (18–64 years), and individuals with primary or less than primary education (aOR=2.76; 95% CI: 1.44–5.28). Of the smokers, 97.2% were daily consumers of whom 44.9% had medium nicotine dependence. Of all smokers, three-quarters expressed a wish to quit, and one-quarter admitted to consuming tobacco during hospitalization.

**CONCLUSIONS:**

Our findings indicate the need to offer smoking cessation interventions among hospitalized patients in all units and service areas, to avoid infringements and increase patient safety, hospital efficiency, and improve clinical outcomes. Hospitalization represents a promising window for initiating smoking interventions addressed to all patients admitted to smoke-free hospitals, specially after applying a smoke-free campus ban.

## INTRODUCTION

Tobacco consumption is responsible for one-sixth of the 6 million annual deaths caused by non-communicable diseases worldwide, including cardiovascular diseases, chronic respiratory diseases and cancer^1^. In 2005, the Framework Convention on Tobacco Control (FCTC) promoted several policies to tackle the tobacco epidemic. Of these policies, Article 8 proposed smoking bans and Article 14 directed countries to implement effective programs to assist individuals in quitting tobacco use^2^. In addition, the FCTC asserted that health organizations and healthcare professionals should act as examples in controlling tobacco consumption, championing compliance with the law, and providing smoking cessation aids^3^.

The scientific literature has shown that tobacco control policies adopted in healthcare organizations have mainly achieved changes in organizations and workers. These changes include reductions in smoking prevalence, increases in the number of attempts to quit smoking among health professionals^4-6^, and increases in the number of tobacco cessation interventions available^7,8^. However, only a small number of these studies have evaluated the impact of these measures on patients that consume tobacco. Smoking among hospitalized patients continues to be a widely neglected problem. Hospitalized patients exhibit a high smoking prevalence and frequent infringements of smoke-free policies^9-11^. In Spain, the tobacco epidemic is at Stage IV, according to the cigarette epidemic model. This model shows a marked downturn in smoking prevalence among men and women and a decline of deaths attributable to smoking among men, but an increase among women^12^. According to the latest Spanish National Health Survey, tobacco consumption among adults (≥15 years old) has dropped from 38.7% in 2001^13^ to 25.4% in 2014^14^. Despite this important decline, tobacco-related diseases continue to cause 15.2% of the total mortality in Spain^15^. Approximately 5 million individuals receive medical or surgical treatment in acute-care hospitals annually in Spain^16^, of whom about 1 million are smokers.

In 2011, Spain established one of the most comprehensive tobacco control regulation policies in Europe for healthcare services^17^. The Spanish legislation pioneered an innovative legal framework that banned smoking in indoor and outdoor areas of acute-care hospitals. Responsibility was given to hospital management and health regional administrators to provide effective smoking cessation interventions, including counseling and pharmacological therapy, to address the needs of hospitalized patients that consumed tobacco^18^.

Once the new Spanish legislation was established, the opportunity arose for evaluating the impact of tobacco control laws among inpatients and for assessing determinants of smoking during hospitalization. The ultimate goal of these studies is to improve our understanding of the needs of patients that attempt to quit smoking. This information would facilitate the design of tobacco cessation services and interventions for initiating attempts to quit smoking in a supportive, smoke-free environment. Therefore, this study aimed to assess the prevalence, determinants, and patterns of smoking before and during hospital admission, in 13 hospitals in the province of Barcelona, Spain.

## METHODS

### Design

In this multi-center cross-sectional study, we conducted a survey of a random sample of hospitalized patients admitted to 13 acute-care hospitals in Barcelona province (Northeast Spain), which comprises an area of 7.733 km^2^ of 5.5 million inhabitants. Hospitals were selected by convenience from the 38 acute-care hospitals that belonged to the Catalan Network for Smoke-free Hospitals (XCHsF, www.xchsf.com) in Barcelona province.

### Participants

Volunteers participated in the study. Participants were hospitalized adults (≥18 years), conscious and oriented in space, time, and person, with a stay ≥24 hours. All participants provided informed consent. Patients that were hospitalized from the emergency room and intensive care units were excluded.

Sample sizes were calculated to be representative of each hospital and, after weighting, representative of all of Catalonia. Estimation of the sample size for each hospital took into account the total number of acute-care beds and the available regional smoking prevalence in Catalonia (29.4%)^19^. The required sample size was 1034 subjects, assuming a precision of ± 5% and an error of 5%. The calculation was performed with Statcalc in EpiInfo, version 6.0.4 (Centers for Disease Control and Prevention, Atlanta, US).

In each hospital, individuals were randomly selected from the daily updated admission list. The randomization system for selecting participants was based on four steps: 1) the number of cases in each hospital was divided by the number of beds, 2) a random number was selected between one and the value attained in step one, 3) the first selection was the case number that matched the number chosen in step two, 4) the next case was selected by adding the selected number in step two to the number obtained in step one. For example, for a hospital with 350 beds, the sample size needed to account for 70 patients. Next, we divided the bed number (350) of the hospital by the sample number (70) of participants selected, i.e. 350/70=5. We chose a random number between 1 and 5 (e.g. 3). We began selecting the participants with that number (e.g. 3) and the 5th case patients were invited to participate. Thus, our invitation list became: 3, 8, 13, 18, and so on.

**Figure 1:**
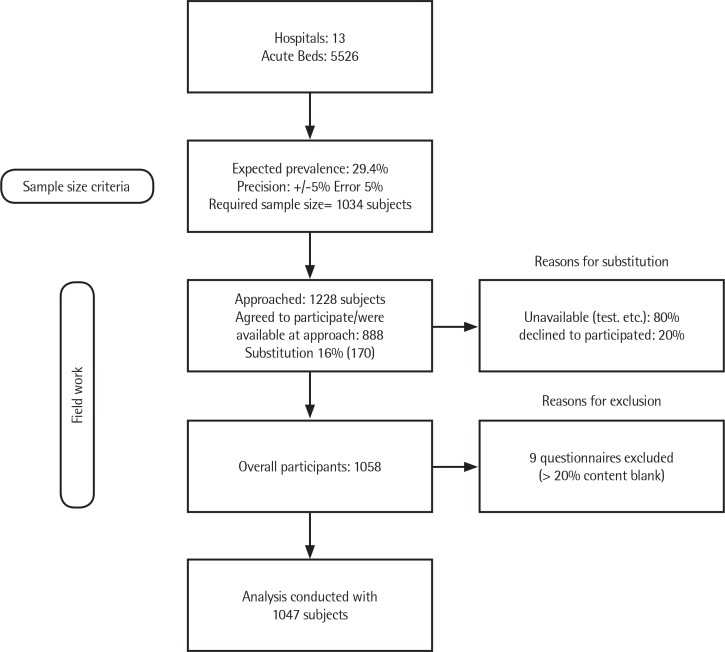
Flowchart of the recruitment process

When a selected patient corresponded to an empty bed or a patient that was unavailable (for instance, in a test) or when the patient declined to participate, we invited the next patient on the list that fulfilled the inclusion and exclusion criteria. In each survey, this type of substitution accounted for less than 16% of the corresponding sample. Thus, we approached 1228 subjects in total, 888 accepted to participate and 170 were replaced (80% were not available and 20% declined to participate at the time of the interview). From the overall 1058 questionnaires obtained, 9 were excluded from the analysis because >20% of their content was blank (Figure 1).

### Data collection

We used an *ad hoc* questionnaire developed by an expert working group from the XCHsF (www.xchsf.com). This questionnaire was composed of 86 questions exploring several dimensions, however for the purpose of this study the main outcome variable was tobacco consumption that was defined according to WHO criteria^20^. Respondents were classified into three categories: 1) current smoker, defined as a person that smoked regularly, either daily (at least one cigarette per day – CPD) or occasionally (less than one CPD) at the time of the survey; 2) non-smoker, defined as a person that had never smoked or had smoked less than 100 cigarettes in his/her lifetime; and 3) former smoker, defined as a person that smoked in the past, but had quit at least 6 months prior to the study. Subjects were asked about their tobacco consumption before and during their hospital admission.

To assess smoking patterns, current and former smokers were asked at what age they had started smoking and the number of cigarettes smoked per day. Among current smokers, we assessed what type of tobacco product they used (cigarettes, roll your own – RYO, electronic cigarettes, cigars, or pipe); the number of cigarettes smoked per day (CPD; classified as <10, 10–19, and ≥20); and the time to the first cigarette after waking (≤5, 6–30, 31–60, >60 min). Nicotine dependence was assessed with the Heavy Smoking Index (HSI), a six-point scale calculated from the number of cigarettes smoked per day and the time to the first cigarette after waking. The HSI scores were categorized into three levels of nicotine dependence: low (0–2), medium (3–4), and high (5– 6)^21^. Withdrawal symptoms were assessed according to DSM-IV categories. Smokers also reported whether they had abstained from smoking during hospitalization (yes/no). We measured exhaled carbon monoxide (CO) to validate abstinence with a CO-oximeter. The cutoff value for active tobacco consumption was set at 6 particles per million (ppm)^22^. We assessed the readiness to quit with the Prochaska and DiClemente change model stages that include: pre-contemplative, contemplative, active, maintenance, and relapse^23^.

The main descriptive variables relating to patients were: 1) sociodemographics, such as sex and age (classified as <45 years, 45–64 years, >64 years, based on the median distribution); 2) educational level (less than primary, primary, high school, and university); 3) occupation (employed, unemployed, retired, other – students and homecare roles); 4) partner’s smoking status (no partner, non-smoking partner, smoking partner); 5) self-perceived health status (excellent, very good, good, adequate, or poor); 6) independence index, according to the Barthel index (100 independent, <100 dependent); and 7) comorbidities.

In addition, we explored the following variables relating to hospitals: 1) the level of technology of the center (general or high-technology hospital); 2) type of ward (the original ward was noted, but later categorized as surgical, medical-surgical, or medical); 3) number of beds (≤300, >300); 4) smoking prevalence among health professionals (≤30%, >30%, based on records in the Catalan Network for Smoke-free Hospitals files (XCHsF); 5) smoking cessation program at the hospital (yes, no); 6) accreditation level, according to XCHsF standards (gold, silver, bronze, and member).

The questionnaire had been tested previously with a pilot sample of participants in one hospital. During the pilot testing a couple of questions were slightly changed mainly because interviewers mentioned that patients did not understand them. For example, instead of employing the term ‘e-health’ we asked current smokers and former smokers whether they have used webs, apps, and other Internet resources for quitting. We collected data with a computer-assisted personal interview (CAPI) system. Trained interviewers conducted the interviews face-to-face, from May 2014 to May 2015.

### Ethical considerations

The study protocol was approved by the Ethics Committee of the Hospital Universitari de Bellvitge (PR234/11). All participants provided written consent to participate.

### Data analysis

Descriptive analyses were performed on participant and center characteristics. Tobacco consumption prevalence was computed overall, according to sex, and for each center. Direct standardization was used to control for age. The reference population was all the participants in the study, and the following age groups were used for standardization: 18–29, 30–39, 40–49, 50–59, 60–69, and ≥70 years.

The main outcome variable was smoking status (current smoker, former smoker, and non smoker). A bivariate analysis was carried out with logistic regression to estimate the association between the main outcome variables and the sociodemographic and center characteristics. To identify the main determinants that characterized a smoker and/ or former smoker, we fitted polytomous logistic regression models. Results are presented as crude odds ratio (cOR) or adjusted OR (aOR), as a measure of association. We used a multilevel logistic regression model, due to the variability in the prevalence of tobacco use among centers. Statistical significance was set at p<0.05. Analyses were conducted with SPSS version 21 and STATA version 13. The weights from the sample design were applied to all calculations.

## RESULTS

### Sociodemographic data

The final sample comprised 1047 subjects. The number of participants per hospital ranged from 41 to 205. Table 1 shows the participant sociodemographic characteristics and the main variables. In brief, half of participants were men, predominantly >64 years old (45.6%). The majority had less than primary or primary education (66.1%), were retired (50.6%), and with a nonsmoking partner (53.8%). Before hospitalization, most declared that they were in good (43.2%) or adequate to poor health (41.1%), and most (78.0%) completed daily activities independently (without assistance).

**Table 1:** Smoking status according to variables that represent patient characteristics (Hospitals of Barcelona Province, 2014–2015)

	*Overall*			*Current smokers*			*Former smokers*			*Non-smokers*		

	*n*	*%^1^*	*95% CI*	*n*	*%^2^*	*95% CI*	*n*	*%^2^*	*95% CI*	*n*	*%^2^*	*95% CI*
**Overall**	1047	100	–	215	20.5	(18.1 – 23.0)	346	33.1	(30.2 – 35.9)	486	46.4	(43.4 – 49.4)
**Sex**												
Female	520	49.7	(46.6 – 52.7)	70	13.5	(10.5 – 16.4)	95	18.3	(14.9 – 21.6)	355	68.2	(64.3 – 72.3)
Male	527	50.3	(47.3 – 53.4)	145	27.5	(23.7 – 31.3)	251	47.6	(43.4 – 51.9)	131	24.9	(21.2 – 28.5)
**Age groups (years)**												
<45	255	24.4	(21.8 – 27.0)	81	31.8	(26.1 – 37.5)	49	19.2	(14.4 – 24.1)	125	49.0	(42.9 – 55.2)
45–64	314	30.0	(27.2 – 32.8)	96	30.6	(25.5 – 35.7)	122	38.8	(33.5 – 44.2)	96	30.6	(25.5 – 35.7)
>64	478	45.6	(42.6 – 48.7)	38	7.9	(5.5 – 10.4)	175	36.7	(32.3 – 40.9)	265	55.4	(51.0 – 59.9)
**Education**												
Less than primary	360	34.5	(31.6 – 37.4)	46	12.8	(9.3 – 16.2)	113	31.4	(26.6 – 36.2)	201	55.8	(50.7 – 61.0)
Primary	330	31.6	(28.8 – 34.4)	95	28.8	(23.9 – 33.7)	111	33.6	(28.5 – 38.7)	124	37.6	(32.4 – 42.8)
High School	213	20.4	(18.0 – 22.8)	55	25.8	(19.9 – 31.7)	68	31.9	(25.7 – 38.2)	90	42.3	(35.6 – 48.9)
University	141	13.5	(11.4 – 15.6)	19	13.5	(7.8 – 19.1)	54	38.3	(30.3 – 46.3)	68	48.2	(40.0 – 56.5)
**Occupation**												
Employed	321	30.7	(27.9 – 33.5)	101	31.5	(26.4 – 36.5)	92	28.7	(23.7 – 33.6)	128	39.8	(34.5 – 45.2)
Unemployed	77	7.4	(5.8 – 8.9)	30	38.9	(28.1 – 49.9)	17	22.1	(12.8 – 31.3)	30	39.0	(28.1 – 49.9)
Retired	532	50.7	(47.8 – 53.8)	75	14.1	(11.1 – 17.1)	227	42.7	(38.5 – 46.9)	230	43.2	(39.0 – 47.4)
Others	117	11.2	(9.3 – 13.1)	9	7.7	(2.9 – 12.5)	10	8.5	(3.5 – 13.6)	98	83.8	(77.1 – 90.4)
**Partner's smoking status**												
No partner	312	29.9	(27.1 – 32.6)	74	23.7	(19.0 – 28.4)	76	24.4	(19.6 – 29.1)	162	51.9	(46.4 – 57.5)
Non-smoker partner	563	53.8	(50.9 – 56.9)	68	12.1	(9.4 – 14.8)	224	39.8	(35.7 – 43.8)	271	48.1	(44.0 – 52.3)
Smoker partner	170	16.3	(14.0 – 18.5)	72	42.3	(34.9 – 49.8)	46	27.1	(20.4 – 33.7)	52	30.6	(23.7 – 37.5)
**Perceived health status**												
Excellent/Very good	164	15.7	(13.5 – 17.9)	39	23.8	(17.3 – 30.3)	39	23.8	(17.3 – 30.3)	86	52.4	(44.8 – 60.1)
Good	453	43.2	(40.3 – 46.3)	100	22.1	(18.3 – 25.9)	148	32.7	(28.4 – 37.0)	205	45.2	(40.7 – 49.8)
Adequate/Poor	430	41.1	(38.1 – 44.0)	76	17.7	(14.1 – 21.3)	159	37.0	(32.4 – 41.5)	195	45.3	(40.6 – 50.1)
**Barthel Index**												
Dependent (<100)	230	22.0	(19.5 – 24.5)	28	12.2	(7.9 – 16.4)	83	36.1	(29.9 – 42.3)	119	51.7	(45.3 – 58.2)
Independent (100)	817	78.0	(75.5 – 80.5)	187	22.9	(20.0 – 25.8)	263	32.2	(29.0 – 35.4)	367	44.9	(41.5 – 48.3)
Comorbidities*												
Arterial hypertension	431	41.2	(38.2 – 44.1)	56	13.0	(9.8 – 16.2)	166	38.5	(33.9 – 43.1)	209	48.5	(43.8 – 53.2)
Diabetes	270	25.8	(23.1 – 28.4)	33	12.2	(8.3 – 16.1)	106	39.3	(33.4 – 45.1)	131	48.5	(42.6 – 54.5)
Pneumonia	216	20.6	(18.2 – 23.1)	30	13.9	(9.3 – 18.5)	103	47.7	(41.0 – 54.3)	83	38.4	(31.9 – 44.9)
Kidney diseases	201	19.2	(16.8 – 21.6)	24	11.9	(7.5 – 16.4)	93	46.3	(39.4 – 53.2)	84	41.8	(35.0 – 48.6)
Chronic liver diseases	129	12.3	(10.3 – 14.3)	30	23.3	(16.0 – 30.5)	49	38.0	(29.6 – 46.4)	50	38.7	(30.4 – 47.2)
Cancer	256	24.5	(21.8 – 27.1)	45	17.6	(12.9 – 22.2)	105	41.0	(35.0 – 47.0)	106	41.4	(35.4 – 47.4)
Heart diseases	258	24.6	(22 – 27.3)	33	12.8	(8.7 – 16.9)	99	38.4	(32.4 – 44.3)	126	48.8	(42.7 – 54.9)
Cerebrovascular diseases	108	10.3	(8.5 – 12.2)	13	12.0	(5.9 – 18.2)	35	32.4	(23.6 – 41.2)	60	55.6	(46.2 – 64.9)
Respiratory diseases	208	19.9	(17.4 – 22.3)	44	21.2	(15.6 – 26.7)	84	40.3	(33.7 – 47.1)	80	38.5	(31.8 – 45.1)
**Exhaled carbon monoxide**												
CO ≤6 ppm	588	56.2	(53.2 – 59.2)	78	13.3	(10.5 – 16.0)	197	33.5	(29.7 – 37.3)	313	53.2	(49.2 – 57.3)
CO >6 ppm	260	24.8	(22.2 – 27.4)	103	39.6	(33.7 – 45.6)	78	30.0	(24.4 – 35.6)	79	30.4	(24.8 – 36.0)

* Multiple responses allowed. CI: confidence interval. 1 Column %. 2 Row %.

**Figure 2:**
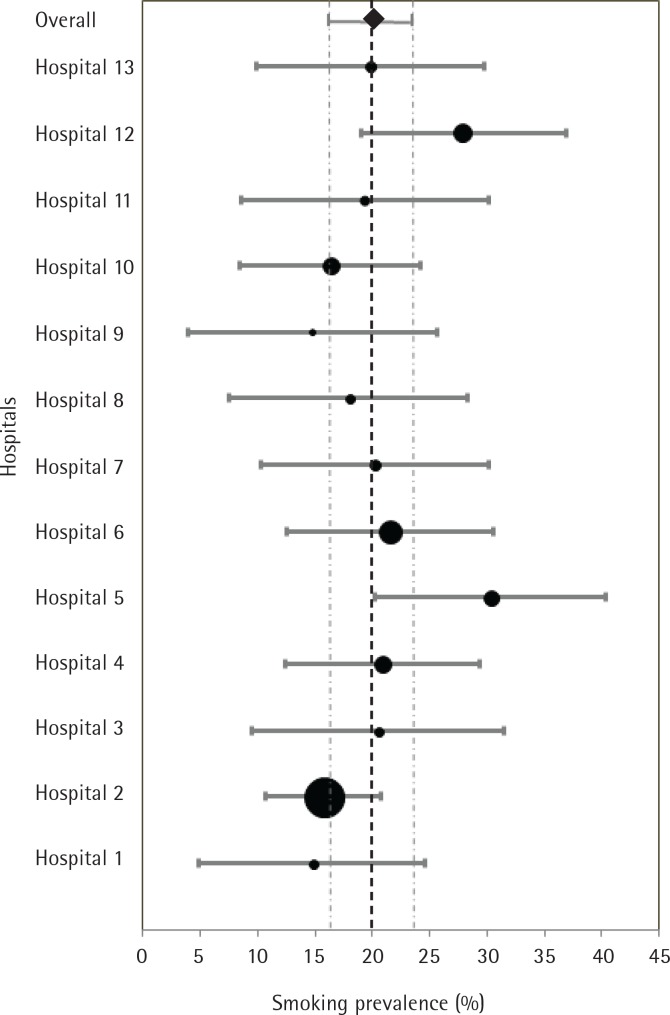
Age-standardized prevalence (%) of smoking, in participant hospitals of Barcelona Province, 2014–2015 (The area of each dot represents the relative weighting of the hospital, in terms of the proportion of participants among the total, bars represent 95% CI, the diamond and the dotted line represent the overall weighted prevalence and 95% CI)

Overall, 20.5% (95% CI: 18.1–23.0) of respondents were current smokers; this proportion varied by hospital, with a range of 14.7 to 30.3% (Figure 2). By sex, 27.5% of men were smokers (95% CI: 23.7– 31.3) and 13.5% were women (95% CI: 10.5–16.4). The highest smoking prevalence was observed in the youngest group, <45 years, for both men and women (men 47.2%, 95% CI: 36.8–57.6; women 23.5%, 95% CI: 17.0–29.9), compared to the middle age group, 45–64 years, (men 38.1%, 95% CI: 31.2–45.0; women 19.2%, 95% CI: 12.3–26.1), and the older age group, >64 years, (men 12.4%, 95% CI: 8.3–16.6; women 3.1%, 95% CI: 0.8–5.3). Among the current smokers, 38.9% were unemployed, 31.5% were employed, and 42.3% had a partner that also smoked.

Former smokers comprised 33.1% of all patients, and significantly different proportions of former smokers were observed between the sexes and among different age groups (Table 1).

### Smoking status according to hospital characteristics

Table 2 summarizes the smoking status of patients in hospitals with different characteristics. The lowest smoking prevalence was observed among medical-surgical wards. In the bivariate analysis, smoking prevalence was not significantly related to any of the explanatory variables we assessed that represented hospital characteristics.

**Table 2:** Smoking status according to variables that represent hospital characteristics (Hospitals of Barcelona Province, 2014–2015)

	*Overall*			*Current smokers*			*Former smokers*			*Non-smokers*		

	*n*	*%^1^*	*95% CI*	*n*	*%^2^*	*95% CI*	*n*	*%^2^*	*95% CI*	*n*	*%^2^*	*95% CI*
**Level of center**												
General hospital	245	23.4	(20.8 – 26.0)	54	22.0	(16.9 – 27.2)	80	32.7	(26.8 – 38.5)	111	45.3	(39.1 – 51.5)
High-technology hospital	802	76.6	(74.0 – 79.2)	161	20.1	(17.3 – 22.8)	266	33.2	(29.9 – 36.4)	375	46.7	(43.3 – 50.2)
**Type of ward**												
Surgical	361	34.5	(31.6 – 37.4)	75	20.8	(16.6 – 25.0)	118	32.7	(27.8 – 37.5)	168	46.5	(41.4 – 51.7)
Medical-Surgical	127	12.1	(10.2 – 14.1)	18	14.2	(8.1 – 20.2)	31	24.4	(16.9 – 31.9)	78	61.4	(53.0 – 69.9)
Medical	559	53.4	(50.4 – 56.4)	122	21.9	(18.4 – 25.2)	197	35.2	(31.3 – 39.2)	240	42.9	(38.8 – 47.0)
**Number of beds**												
≤300	541	51.7	(48.6 – 54.7)	111	20.5	(17.1 – 23.9)	176	32.5	(28.6 – 36.5)	254	47.0	(42.7 – 51.2)
>300	506	48.3	(45.3 – 51.4)	104	20.6	(17.0 – 24.1)	170	33.6	(29.5 – 37.7)	232	45.8	(41.5 – 50.2)
HP smoking prevalence												
<30%	801	76.5	(73.9 – 79.1)	160	20.0	(17.2 – 22.7)	265	33.1	(29.8 – 36.3)	376	46.9	(43.5 – 50.4)
≥30%	246	23.5	(20.9 – 26.1)	55	22.4	(17.2 – 27.6)	81	32.9	(27.1 – 38.8)	110	44.7	(38.5 – 50.9)
**Smoking cessation program**												
Yes	813	77.7	(75.1 – 80.2)	165	20.3	(17.5 – 23.1)	273	33.6	(30.3 – 36.8)	375	46.1	(42.7 – 49.6)
No	234	22.3	(19.8 – 24.9)	50	21.4	(16.1 – 26.6)	73	31.2	(25.3 – 37.1)	111	47.4	(41.0 – 53.8)
**Accreditation level**												
Gold	337	32.2	(29.4 – 35.0)	69	20.5	(16.2 – 24.8)	126	37.4	(32.2 – 42.6)	142	42.1	(36.9 – 47.4)
Silver	317	30.3	(27.5 – 33.1)	63	19.9	(15.5 – 24.3)	91	28.7	(23.7 – 33.7)	163	51.4	(45.9 – 56.9)
Bronze	147	14.0	(11.9 – 16.1)	28	19.0	(12.7 – 25.4)	48	32.7	(25.1 – 40.2)	71	48.3	(40.2 – 56.4)
Member	246	23.5	(20.9 – 26.1)	55	22.4	(17.2 – 27.6)	81	32.9	(27.1 – 38.8)	110	44.7	(38.5 – 50.9)

HP: health professionals. 1 Percentage per column. 2 Percentage per row.

### Smoking patterns among daily smokers before and during hospitalization

Among all smokers, 97.2% were daily smokers (209/215). The majority of male smokers were 45– 64 years (49.6%, 95 CI%: 41.3–58.0); the majority of female smokers were ≤45 years (55.7%, 95 CI%: 44.1–67.4). Both men and women smoked mainly manufactured cigarettes, but nearly one-quarter smoked RYO cigarettes. Other tobacco products (cigars, pipes, and e-cigarettes) were consumed based on anecdotal reports. The majority of smokers consumed exclusively manufactured cigarettes (75.6%), but 12.7% consumed only RYOs, and 11.7% combined these two tobacco products (Table 3).

**Table 3:** Patterns of daily tobacco use, before and during hospitalization (Hospitals of Barcelona Province, 2014–2015)

				*Sex*					
	*Daily smokers (N=209)*			*Men (N=139)*			*Women (N=70)*		

	*n*	*%*	*95% CI*	*n*	*%*	*95% CI*	*n*	*%*	*95% CI*
**Age groups (years)**									
<45	79	37.8	(31.2 – 44.4)	40	28.8	(21.3 – 36.3)	39	55.7	(44.1 – 67.4)
45–64	93	44.5	(37.8 – 51.2)	69	49.6	(41.3 – 58.0)	24	34.3	(23.2 – 45.4)
>64	37	17.7	(12.5 – 22.9)	30	21.6	(14.7 – 28.4)	7	10.0	(3.0 – 17.0)
**Before hospitalization**									
Type of tobacco product consumed*									
Manufactured cigarettes	179	85.6	(80.9 – 90.4)	116	83.5	(77.3 – 89.6)	63	90.0	(83.0 – 97.0)
RYO cigarettes	50	23.9	(18.1 – 29.7)	34	24.5	(17.3 – 31.6)	16	22.9	(13.0 – 32.7)
Electronic cigarettes	2	1.0	(0 – 2.3)	2	1.4	(0 – 3.4)	0	0.0	(0 – 0)
Cigars	14	6.7	(3.3 – 10.1)	14	10.1	(5.1 – 15.1)	0	0.0	(0 – 0)
Pipe	4	1.9	(0.1 – 3.8)	4	2.9	(0.1 – 5.7)	0	0.0	(0 – 0)
**Combination of tobacco products**									
Only manufactured cigarettes	155	75.6	(69.7 – 81.5)	101	74.8	(67.5 – 82.1)	54	77.1	(67.3 – 87.0)
Only RYO cigarettes	26	12.7	(8.1 – 17.2)	19	14.1	(8.2 – 19.9)	7	10.0	(3.0 – 17.0)
Both	24	11.7	(7.3 – 16.1)	15	11.1	(5.8 – 16.4)	9	12.9	(5.0 – 20.7)
**Number of cigarettes per day (manufactured + RYO)**									
<10	54	26.5	(20.4 – 32.5)	31	23.2	(16.0 – 30.3)	23	32.9	(21.9 – 43.9)
10–19	90	44.1	(37.3 – 50.9)	57	42.5	(34.2 – 50.9)	33	47.1	(35.4 – 58.8)
≥20	60	29.4	(23.2 – 35.7)	46	34.3	(26.3 – 42.4)	14	20.0	(10.6 – 29.4)
**Time (min) to first cigarette after waking**									
≤30	152	72.7	(66.7 – 78.8)	98	70.5	(62.9 – 78.1)	54	77.1	(67.3 – 87.0)
>30	57	27.3	(21.2 – 33.3)	41	29.5	(21.9 – 37.1)	16	22.9	(13.0 – 32.7)
**Heavy Smoking Index**									
High	39	18.7	(13.4 – 23.9)	28	20.2	(13.5 – 26.8)	11	15.7	(7.2 – 24.2)
Medium	94	44.9	(38.2 – 51.7)	59	42.4	(34.2 – 50.7)	35	50.0	(38.3 – 61.7)
Low	76	36.4	(29.8 – 42.9)	52	37.4	(29.4 – 45.5)	24	34.3	(23.2 – 45.4)
**During hospitalization**									
**Would you like to quit smoking if you could do it easily?**									
Yes	153	75.7	(69.8 – 81.7)	106	77.4	(70.4 – 84.4)	47	72.3	(61.4 – 83.2)
No	49	24.3	(18.3 – 30.2)	31	22.6	(15.6 – 29.6)	18	27.7	(16.8 – 38.6)
**How much interest do you have in quitting now?**									
None	43	21.3	(15.6 – 26.9)	25	18.7	(12.1 – 25.3)	18	26.5	(16.0 – 37.0)
Some	36	17.8	(12.5 – 23.1)	24	17.9	(11.4 – 24.4)	12	17.6	(8.6 – 26.7)
Sufficient	46	22.8	(17.0 – 28.6)	31	23.1	(16.0 – 30.3)	15	22.1	(12.2 – 31.9)
High	77	38.1	(31.4 – 44.8)	54	40.3	(32.0 – 48.6)	23	33.8	(22.6 – 45.1)
**Cessation contemplation status**									
Precontemplative	77	38.5	(31.8 – 45.2)	47	34.8	(26.8 – 42.9)	30	46.2	(34.0 – 58.3)
Contemplative	55	27.5	(21.3 – 33.7)	37	27.4	(19.9 – 34.9)	18	27.7	(16.8 – 38.6)
Ready	35	17.5	(12.2 – 22.8)	26	19.3	(12.6 – 25.9)	9	13.8	(5.4 – 22.2)
Active	33	16.5	(11.4 – 21.6)	25	18.5	(12.0 – 25.1)	8	12.3	(4.3 – 20.3)
**Tobacco use during hospitalization**									
No	157	75.8	(69.2 – 82.5)	105	76.6	(68.5 – 84.7)	52	74.3	(62.4 – 86.2)
Yes	50	24.2	(17.5 – 30.8)	32	23.4	(15.3 – 31.5)	18	25.7	(13.8 – 37.6)
**Exhaled carbon monoxide (CO) ppm level**									
CO ≤6	73	41.7	(34.4 – 49.0)	44	37.0	(28.3 – 45.6)	29	51.8	(38.7 – 64.9)
CO >6	102	58.3	(51.0 – 65.6)	75	63.0	(54.4 – 71.7)	27	48.2	(35.1 – 61.3)

* Multiple responses allowed. RYO: roll your own cigarettes.

Before hospitalization, 44.1% of daily smokers consumed 10–19 CPD, and 72.7% consumed their first cigarette within the first 30 min of waking. Thus, 44.9% of smokers had an intermediate level of nicotine dependence according to the HSI. High levels of nicotine dependence were more frequently observed among male smokers than among female smokers, but the difference was not statistically significant (Table 3).

A total 75.7% of daily smokers expressed their wish to quit smoking, if it could be done easily during hospitalization. Among all smokers, 38.1% had high interest in quitting now, and 21.3% had no interest in quitting. Men were more interested in quitting than women (Table 3). Among daily smokers, 24.2% admitted to consuming tobacco during hospitalization, and the proportion was similar between men and women. When analysing their length of stay and smoking during hospitalization, we observed that 31.6% of smokers with 1-day stay smoked during hospitalization versus 19.8% of those who were interviewed in their 2nd to 5th day of stay, and 26.5% of those with >5 days of stay (p=0.417). The average number of cigarettes consumed per day was 3 (range=1 to 20). Smokers who consumed tobacco did it 15.5% of the times inside the hospital (in their room, wc, stairs, terraces), 63.5% in outdoor areas belonging to the hospital (entrances, gardens, parkings, etc), and 21.0% outside the perimeter of the hospital. Smokers who consumed tobacco during hospitalization did not differ from those who remained abstinent from smoking, based on sex, age, nicotine dependence, and whether the hospital provided smoking cessation services. The percentage of smokers with exhaled carbon monoxide levels >6 ppm was higher among male smokers than among female smokers (63.0% vs 48.2%, p=0.069) It is known, that smokers who remain abstinent for more than 6 days might have lower CO levels^22^. Our results showed that 73.9% of smokers (with more than 5 days stay) who admitted to having smoked during hospitalization had a positive CO (> 6ppm) in contrast to 34.9% of those who remained abstinent (p=0.001).

### Smoking patterns and sociodemographic characteristics of former smokers

Male former smokers were mostly >64 years, and female former smokers were significantly younger (45–64 years; p≤0.001, Table 4). Former smokers were more likely to have smoked daily, rather than occasionally, but a higher percentage of women than men had smoked occasionally. Among former daily smokers, we observed differences between the sexes. For example, the majority of men previously consumed ≥20 CPD (70.9%, 95% CI: 64.7–77.1) and a minority of women previously consumed ≥20 CPD (37.5%, 95% CI: 25.6–49.4; p<0.05). About 74.1% of former smokers had attempted to quit 1 or 2 times before finally quitting. The two most reported reasons for quitting were their own personal decision and health concerns. Among former smokers, 87.3% did not use any resources to assist them in quitting, and 13.5% used a pharmacological treatment to quit (Table 4).

**Table 4:** Smoking pattern and sociodemographic characteristics among former smokers (n=346) (Hospitals of Barcelona Province, 2014–2015)

				*Sex*					
	*Former smoker*			*Men*			*Women*		

	*n*	*%*	*95% CI*	*n*	*%*	*95% CI*	*n*	*%*	*95% CI*
**Age groups (years)**									
<45	49	14.2	(10.5 – 17.8)	17	6.8	(3.7 – 9.9)	32	33.7	(24.2 – 43.2)
45–64	122	35.3	(30.2 – 40.3)	81	32.3	(26.5 – 38.1)	41	43.1	(33.2 – 53.1)
>64	175	50.5	(45.3 – 55.8)	153	60.9	(54.9 – 67.0)	22	23.2	(14.7 – 31.6)
**Daily consumption**									
No (occasionals)	47	13.6	(9.7 – 17.5)	20	8.0	(4.5 – 11.5)	27	28.4	(17.7 – 39.1)
Yes	299	86.4	(82.5 – 90.3)	231	92.0	(88.5 – 95.5)	68	71.6	(60.9 – 82.3)
Number of cigarettes per day (manufactured + RYO) *									
<10	40	14.8	(10.6 – 19.1)	19	9.2	(5.3 – 13.2)	21	32.8	(21.3 – 44.3)
10–19	60	22.2	(17.3 – 27.2)	41	19.9	(14.5 – 25.4)	19	29.7	(18.5 – 40.9)
≥20	170	63.0	(57.2 – 68.7)	146	70.9	(64.7 – 77.1)	24	37.5	(25.6 – 49.4)
**Number of quit attempts**									
1–2	253	74.1	(69.5 – 78.8)	178	72.0	(66.5 – 77.7)	75	79.8	(71.7 – 87.9)
3–5	66	19.4	(15.2 – 23.5)	52	21.1	(16.0 – 26.1)	14	14.9	(7.7 – 22.1)
≥6	22	6.5	(3.8 – 9.1)	17	6.9	(3.7 – 10.0)	5	5.3	(0.8 – 9.9)
Reasons for quitting **									
Doctor/nurse recommendation	110	31.8	(26.9 – 36.7)	93	26.9	(22.2 – 31.5)	17	4.9	(2.6 – 7.2)
Tobacco use annoyances	134	38.7	(33.6 – 43.9)	118	34.1	(29.1 – 39.1)	16	4.6	(2.4 – 6.8)
Health concerns	175	50.6	(45.3 – 55.8)	141	40.8	(35.6 – 45.9)	34	9.8	(6.7 – 13.0)
Decrease of my physical performance	75	21.7	(17.3 – 26.0)	66	19.1	(14.9 – 23.2)	9	2.6	(0.9 – 4.3)
Familial pressure	71	20.5	(16.3 – 24.8)	47	13.6	(10.0 – 17.2)	24	6.9	(4.3 – 9.6)
Personal decision	215	62.1	(57.0 – 67.2)	154	44.5	(39.3 – 49.7)	61	17.6	(13.6 – 21.6)
Economic reasons	21	6.1	(3.6 – 8.6)	14	4.0	(2.0 – 6.1)	7	2.0	(0.5 – 3.5)
Health problems (tobacco-related diseases)	15	4.3	(2.2 – 6.5)	12	3.5	(1.5 – 5.4)	3	0.9	(0 – 1.8)
Other reasons	35	10.1	(6.9 – 13.3)	20	5.8	(3.3 – 8.2)	15	4.3	(2.2 – 6.5)
Resources used to quit smoking **									
Professional healthcare	31	9.0	(6.0 – 12.0)	26	7.5	(4.7 – 10.3)	5	1.4	(0.2 – 2.7)
Use of e-health technologies (apps. webs)	0	0.0	(0 – 0)	0	0.0	(0 – 0)	0	0.0	(0 – 0)
Self-help book	7	2.0	(0.5 – 3.5)	5	1.4	(0.2 – 2.7)	2	0.6	(0 – 1.4)
Other	7	2.0	(0.5 – 3.5)	6	1.7	(0.4 – 3.1)	1	0.3	(0 – 0.9)
None	302	87.3	(83.8 – 90.8)	216	62.4	(57.3 – 67.5)	86	24.9	(20.3 – 29.4)
**Pharmacological treatment used**									
Yes	46	13.5	(9.9 – 17.2)	36	14.6	(10.2 – 19.1)	10	10.6	(4.4 – 16.9)
No	294	86.5	(82.8 – 90.1)	210	85.4	(80.9 – 89.8)	84	89.4	(83.1 – 95.6)

* Only daily consumers responded.

** Multiple response

### Predictors associated with smokers and former smokers

Table 5 displays the aOR (adjusted for sex and age) that a current smoker or a former smoker would exhibit characteristics represented by patient-related and hospital-related explanatory variables. Current smokers were more likely to be young men. Tobacco consumption was more likely (aOR=2.76) to be observed among patients with primary or less than primary education levels, compared to patients with a university degree. No other significant relationship emerged between smoking and any of the hospital-related variables studied.

**Table 5:** Univariate and multivariate models of a current smoker or former smoker (Hospitals of Barcelona Province, 2014–2015)

	*Current smoker*				*Former smoker*			

*Descriptive variables*	*cOR*	*95% CI*	*aOR*	*95% CI*	*cOR*	*95% CI*	*aOR*	*95% CI*

*Patient-related characteristics*								
**Sex**								
Male	7.22	(4.88 – 10.68)	7.47	(4.88 – 11.43)	7.11	(5.15 – 9.80)	5.85	(4.16 – 8.22)
Female	1.00		1.00		1.00		1.00	
**Age groups (years)**								
18–29	7.56	(3.63 – 15.77)	8.24	(3.25 – 20.91)	0.48	(0.21 – 1.06)	0.74	(0.30 – 1.84)
30–39	11.07	(5.69 – 21.54)	11.55	(4.84 – 27.55)	1.46	(0.85 – 2.52)	1.63	(0.78 – 3.38)
40–49	13.32	(7.05 – 25.17)	11.48	(5.10 – 25.82)	1.22	(0.70 – 2.11)	1.41	(0.70 – 2.85)
50–59	11.76	(6.23 – 22.20)	10.01	(4.60 – 21.81)	2.35	(1.44 – 3.81)	2.83	(1.52 – 5.29)
60–69	3.50	(1.87 – 6.54)	3.70	(1.93 – 7.09)	1.47	(0.98 – 2.22)	1.58	(1.03 – 2.43)
> 70	1.00		1.00		1.00		1.00	
**Education**								
Primary or less	2.97	(1.61 – 5.49)	2.76	(1.44 – 5.28)	0.73	(0.45 – 1.18)	0.75	(0.46 – 1.24)
High School	1.88	(0.98 – 3.61)	1.45	(0.73 – 2.89)	0.76	(0.44 – 1.30)	0.73	(0.42 – 1.25)
University	1.00		1.00		1.00		1.00	
**Occupation**								
Unemployed	1.21	(0.65 – 2.24)	1.16	(0.59 – 2.29)	0.74	(0.37 – 1.50)	0.93	(0.45 – 1.91)
Retired	1.24	(0.69 – 2.21)	0.96	(0.52 – 1.78)	1.33	(0.77 – 2.27)	1.51	(0.87 – 2.62)
Other	0.34	(0.15 – 0.78)	0.29	(0.12 – 0.67)	0.30	(0.14 – 0.65)	0.35	(0.16 – 0.77)
Employed	1.00		1.00		1.00		1.00	
**Partner's smoking status**								
No partner	2.18	(1.40 – 3.39)	2.07	(1.31 – 3.27)	0.84	(0.58 – 1.21)	0.89	(0.61 – 1.29)
Smoker partner	5.75	(3.48 – 9.52)	6.01	(3.57 – 10.11)	1.53	(0.94 – 2.48)	1.50	(0.91 – 2.47)
Non-smoker partner	1.00		1.00		1.00		1.00	
**Perceived health status**								
Excellent/Very good	1.76	(1.00 – 3.07)			1.67	(1.00 – 2.79)		
Good	1.30	(0.78 – 2.18)			1.30	(0.79 – 2.12)		
Adequate/Poor	1.00				1.00			
**Barthel Index**								
Independent (100)	0.93	(0.54 – 1.57)			0.86	(0.59 – 1.26)		
Dependent (<100)	1.00				1.00			
**Level of center**								
General hospital	1.00				1.00			
High-technology hospital	0.76	(0.50 – 1.16)			1.05	(0.73 – 1.51)		
**Type of ward**								
Surgical	1.00				1.00			
Medical-Surgical	0.58	(0.29 – 1.18)			2.39	(1.26 – 4.54)		
Medical	1.09	(0.74 – 1.63)			1.19	(0.85 – 1.67)		
**Number of beds**								
≤300	1.00				1.00			
>300	1.00	(0.70 – 1.44)			0.98	(0.72 – 1.34)		
**HP smoking prevalence**								
<30%	1.00				1.00			
≥30%	1.51	(0.98 – 2.32)			1.01	(0.70 – 1.46)		
**Smoking cessation program**								
Yes	1.00				1.00			
No	1.11	(0.72 – 1.72)			0.82	(0.57 – 1.19)		
**Accreditation level**								
Gold	1.00				1.00			
Silver	0.75	(0.47 – 1.19)			0.75	(0.50 – 1.11)		
Bronze	0.74	(0.41 – 1.34)			0.74	(0.46 – 1.21)		
Member	1.27	(0.78 – 2.09)			0.86	(0.57 – 1.30)		

HP: health professional, cOR: odds ratio adjusted for age and sex, aOR: odds ratio fully adjusted for all the variables.

The odds ratio of being a former smoker was higher among men (aOR=5.85) compared to women. Smokers were more likely to be 30–69 years old, rather than >70 years old. Former smokers were most frequently patients with a university degree and patients that had retired from working. Patients admitted to medical-surgical wards were more likely to be former smokers than those in surgical wards (aOR=2.39).

## DISCUSSION

This study showed that smoking prevalence was high among hospitalized patients. Our findings indicate that hospitalized smokers comprised mainly males that were <64 years old, had primary or less than primary education, and had a partner with a smoking habit. The majority of smokers exhibited an intermediate nicotine dependence, and were mainly in precontemplative or contemplative stages of quitting. A quarter of smokers consumed tobacco during their hospital stay.

Compared to the Catalan Health Survey (ESCA) results from 2014^24^, adult hospitalized patients (>18 years) showed a lower overall prevalence of smokers than the general population older than 15 years (20.5% vs 25.9%). Nonetheless, this might be due to the mean age of our study sample (hospitalized patients), which corresponded to an aging population. In fact, when comparing age groups, the overall prevalence of current smokers in the middle age group (45–64 years; 30.6%) was similar to that of the Catalan population between 45–54 years (30.5%), but higher than the Catalan population between 55–65 years (21.9%)^24^. When comparing sex and age groups, our study found that 47.2% of men <45 years were current smokers; in the Catalan population, the smoking prevalence was lower among men between 35–44 years (35.2%) and higher among men between 25–34 years (50.0%). Again, no differences were found between our sample and the Catalan population for individuals >65 years (men 12.4% vs 10.9%; women 3.1% vs 4.5%, respectively)^24^. These results suggest that the prevalence of smokers among hospitalized patients was not different from that of the general population. This finding indicates that there is an opportunity for health care services to intervene in young patients that are not chronically ill, when they are treated in acute-care hospitals.

Only two previous studies have monitored smoking consumption in hospitalized populations in Catalonia. The earlier study found a higher overall smoking prevalence (27.8% in 2002 and 30.7% in 2004)^25^, and a later study found a slightly lower smoking prevalence (18.8% in 2006)^26^, compared to the overall prevalence reported here (20.5%). These differences might be due to the differences in timing or design; the earlier studies were conducted 10 years ago and only in one center. In addition, the earlier investigations could have carried some selection bias, because not all hospital wards were included in the sampling strategies. This study is the first to monitor tobacco consumption among hospitalized patients from all wards in acute-care hospitals in our region. In the United States, a recent study that included all patients admitted to a General Hospital over 3 years (2007 to 2010) concluded that 21.1% of patients were smokers, and 18.4% had smoked during their hospital stay^11^. However, although our prevalence of tobacco consumption was similar, we found lower compliance with the smoke-free law than that found by Regan and colleagues^11^. This fact raised two safety concerns in our region. First, smoking may have direct, negative consequences that could delay recovery; and second, smoking could cause a fire, putting others in danger in the complicated context of a hospital. Consequently, hospitals in Catalonia should improve their communications about the smoke-free campus policy and about the risks of smoking during hospitalization. In addition, hospitals should monitor the implementation of the smoke-free compliance on a yearly basis.

In Catalonia, there are around 972995 annual hospitalizations^27^. According to our study, 20.5% of acute-care patients smoked, and of these, the majority expressed some or a high interest in quitting. Extending these proportions to all hospitalizations suggests that about 157000 hospitalized smokers might be motivated to quit annually. In addition, as mentioned above, our profile of smokers in general hospitals indicates mainly young men. Thus, our data supports the notion that hospitalization could serve as a promising window of opportunity for approaching smokers and encouraging cessation^28^. This opportunity has the advantages, in the context of a comprehensive smoke-free hospital, that smokers are obliged to abstain at least temporarily from tobacco use and that they are in regular contact with health professionals. A previous study found that between 60–70% of inpatients had attempted to quit smoking while they were hospitalized^29^. In the present study, about three-quarters of smokers expressed a wish to quit smoking, and about one-third was ready to undertake an attempt. Therefore, hospitalization could provide a unique opportunity for identifying and engaging smokers, initiating cessation treatments, and facilitating appropriate follow-up and support procedures^30^.

A previous meta-analysis showed that smoking cessation programs were effective when they began during a hospital stay, regardless of the reason for admission, and when nicotine replacement therapy (NRT) was offered and a follow-up visit was provided at one month after discharge^30^. Therefore, health providers, should offer effective smoking cessation assistance to hospitalized smokers, regardless of the diagnosis or hospital unit, as shown in our study^3^.

Internationally, several health organizations have adopted the 5As intervention model for smoking cessation proposed by evidence-based guidelines^31^. This model is based on five steps: 1) Ask patients about smoking at every visit; 2) Advise all tobacco users to quit; 3) Assess smoker’s willingness to try to quit; 4) Assist smoker’s efforts with treatment and referrals; and 5) Arrange follow-up contacts to support cessation efforts^31^. However, deficiencies persist in implementing smoking cessation interventions as part of routine practices in hospital settings^32^. One situation recognized to render smoking cessation interventions suboptimal is when the health professional is a smoker^33,34^. For instance, it was found that nurses that smoked were less likely to advise their patients to quit and less willing to arrange smoking cessation follow-up appointments^34^. In addition, a recent study conducted among health professionals in Catalonia showed that the clinical healthcare workers did not perform the 5As completely^35^. The main barriers were: smoking cessation was considered not part of their job, lack of familiarity with practical guidelines, lack of previous positive experiences, and lack of organizational support^35^.

To improve smoking cessation interventions, several hospitals have taken numerous actions, including providing training to health professionals and requesting compulsory performance indicators^30,36,37^. Training has been strongly associated with higher levels of confidence, more frequent interventions, and fewer barriers to providing cessation services^38^.

In Catalonia, several actions have been undertaken to implement tobacco control interventions in hospitals, beyond the legislative framework^39^. In 2000, the XCHsF promoted a ‘tobacco control hospital model’ that implemented organizational and cultural changes^39^. This model required the organization to make a commitment to adopting, integrally and progressively, a series of ten standards. This organizational change involved creating a policy working group that was integrated into the hospital management team and included key individuals in the institution (champions). This working group was responsible for designing the tobacco-control policy, scaling it down, and ensuring that it was properly communicated, monitored, and evaluated^38^. Thus, the working group must clearly communicate the policies to the other staff members, the patients, and the community. Currently, the tobacco control champions have shown extraordinary interest in the adoption, implementation, and evaluation of activities^40,41^. Because training is a key factor in the sustainability of these programs, the XCHsF has offered in-person and online training, which takes place every year and aims to reach a broad number of hospital workers^42^.

The large prevalence of smokers with medium and high nicotine dependences (63.6% of smokers) has provided a strong rationale for offering drug treatment, such as nicotine replacement treatment (NRT). Currently, the majority of Catalan hospitals have implemented this therapeutic aid^40^. Clearly, the impact of these measures on increasing health care professionals’s interventions in providing tobacco cessation, patient smoking prevalence and cessation rates should be tested in future studies.

### Limitations

This study has several limitations. First, it is a cross-sectional survey, and thus, our results cannot lead to any conclusions about direct causal effects; instead, our results only indicate associations. Second, this study relied on self-reported responses; however, tobacco consumption was verified with a COoximeter. Third, we excluded patients admitted into emergency units and critical care units; thus, our sample might not be representative of all patients admitted into Catalan hospitals. In cases where admission to those units was related to smoking, omission of these data could lead to a bias in our results towards a lower prevalence of smoking. However, we implemented this exclusion criterion to assure the validity of our information, because all our patients were conscious in space and time. Fourth, due to our selection of hospitals by convenience, we could have introduced a selection bias, because it is possible that we chose only hospitals with the most interest in smoking cessation practices. However, that bias would have led to an underestimation of the current smoking problem in Catalan hospitals in this study. Nonetheless, this study was the first in Spain to explore smoking prevalence among hospitalized patients in multiple hospitals and units, with a large, representative sample size.

## CONCLUSIONS

Overall, 20.5% of hospitalized patients were current smokers, over 60% of smokers had medium or high nicotine dependences, and one-quarter smoked during hospitalization. Moreover, we found that about 75.7% of smokers expressed a wish to quit smoking, and half of them were ready or in a complentative stage to undertake an attempt. Our findings indicate that there is a need to initiate smoking cessation interventions among patients in all units and service areas during hospitalization. It is necessary to provide adequate therapeutic support and routine monitoring of patient cravings for cigarettes. The implementation of cessation interventions could avoid infringements of smoke-free policies and improve patient safety, hospital efficiency, and clinical outcomes. Future investigations should focus on testing the effectiveness of smoke-free policies combined with smoking cessation interventions initiated in hospitals. In addition, our findings indicate that these interventions should mainly focus on young patients that are not chronically ill. This study suggests that hospitalization represents a promising window for initiating smoking interventions addressed to all patients, particularly for patients who are men and without chronic diseases. Finally, health administrations, hospitals, and community services should work together to facilitate the initiation of smoking cessation treatments for patients admitted to smoke-free hospitals, especially after applying smoke-free campus bans.
